# Chronic Complications of Diabetes: Prevalence, Prevention, and Management

**DOI:** 10.3390/jcm13237001

**Published:** 2024-11-21

**Authors:** Ilias N. Migdalis

**Affiliations:** Diabetes Centre, Lefkos Stavros Hospital, 11528 Athens, Greece; ilianmig@otenet.gr

The prevalence of diabetes, especially of type 2 diabetes (T2D), is increasing globally, driven mainly by behavioral and societal factors, related to obesity, nutrition, and physical activity [1]. Based on the International Diabetes Federation (IDF) Atlas 2021, it is estimated that 537 million people have diabetes; this number is expected to reach 643 million by 2030 and 783 million by 2045 [2].

They are, in general, divided into micro- and macrovascular complications. Microvascular complications are specific to diabetes mellitus but occur irrespective of the cause of diabetes. On the other hand, macrovascular complications are more prevalent in diabetes but are not specific to it. They may occur at a younger age in diabetic patients. Microvascular complications include neuropathy, retinopathy, and nephropathy, while macrovascular disease affects the coronary arteries, cerebral circulation, and peripheral arteries [3,4]. Hyperglycemia plays a role in predisposing to macrovascular disease but is critical in the development of microvascular disease [5]. However, it is clear that this classification needs to be revised since there are many diabetic complications that cannot be placed in the two categories. For example, it has been acknowledged that diabetes mellitus, regardless of whether T1D or T2D, is associated with an increased risk of fracture [6]. Another aspect is that a variety of musculoskeletal conditions have been associated with diabetes mellitus, including several disorders affecting the hands, such as limited joint mobility and carpal tunnel syndrome; the shoulders, such as frozen shoulder; and several other conditions. These problems are important to recognize because of their impact on life, particularly as many respond to treatment [7]. Additionally, there are other complications, such as cognitive dysfunction and periodontitis, that cannot be placed in the established classification [8,9].

Yu et al. [10] proposed a new classification. They separated the chronic complications of diabetes into pathological categories of vascular tissues, parenchymal tissues, and hybrid tissues. Parenchymal tissue complications affect mainly nonvascular organs, while a large number of complications in diabetes are the result of both vascular and parenchymal tissues.

Many factors contribute to the initiation and progression of chronic complications of diabetes. Persistent hyperglycemia over many years is the principal underlying cause of the microvascular complications of diabetes. It is also an independent risk factor for the development of macrovascular coronary artery disease. The UKPDS study of T2D showed precisely the increasing hazard in relation to continuously rising HbA1c levels, without any specific threshold point, and then demonstrated the benefits of tight control [11,12]. In all the cases, in order to deduce both micro- and macrovascular complications, the rule must be the multifactorial intervention [13]. While there are known familial trends in the development of some diabetic complications, notably nephropathy, the many attempts to discover genetic markers of susceptibility to particular complications have so far failed to identify any specific variants to help patients. On the other hand, the number of protective factors is growing. These protective factors are more or less equally significant in modulating the development of diabetic complications with the risk factors. However, in clinical practice, there is a gap between the risk and protective factors [14,15]. The complications of diabetes are greatly feared by patients, parents, and other family members. The ever-present threats of blindness and amputations can cause lifelong anxiety. From the doctors’ point of view, diabetes mellitus is no longer viewed as a disease of sugar alone and a more holistic approach is required. So, the management of a diabetic patient should comprise managing, apart from the blood glucose, all the other modifiable risk factors such as hypertension, dyslipidemia, and cessation of smoking.

The Special Issue, “Chronic Complications of Diabetes: Prevalence, Prevention, and Management”, covers a wide field of chronic complications. Atkas [Contribution 1] compares the prognostic nutrition index levels of the subjects with T2D with and without microvascular complications to those of healthy subjects. Tanase et al. [Contribution 2] discuss the hypothetical link across branched-chain amino acid metabolism, insulin resistance, T2D, and its microvascular complications. Dweib and El Sharif [Contribution 3] carried out a study investigating the factors associated with diabetes-related microvascular complications in primary health care settings. Chang et al. [Contribution 4] show that the toe-brachial index has a better yield of detection of peripheral vascular disease compared to the ankle-brachial index among patients with T2D and chronic kidney disease (CKD). Chwal et al. [Contribution 5] provide evidence that glucose and blood pressure control reduce mortality in T2D. Chiu et al. [Contribution 6] conducted a study on sudomotor function in non-dialysis CKD patients. Braha et al. [Contribution 7] share insights for the prevention of intraocular pressure in T2D. Albai et al. [Contribution 8] discuss the impact of vitamin D levels on the healthy status of T2D. Finally, Giannoulaki et al. [Contribution 9] discuss the use of advanced hybrid closed-loop systems in a pregnant woman with type 1 diabetes mellitus.

It is estimated that people with diabetes die, on average, 6 years earlier than people without diabetes [16,17]. The chronic complications of diabetes contribute to this average reduction in life expectancy, highlighting the need to understand and develop interventions to prevent or delay the complications. We hope that this Special Issue will offer new information to the readers and improve their understanding on the topic. Finally, I sincerely thank all the authors for their contribution.
